# In Vitro Selection of High-Level Beta-Lactam Resistance in Methicillin-Susceptible *Staphylococcus aureus*

**DOI:** 10.3390/antibiotics10060637

**Published:** 2021-05-26

**Authors:** Vladimir Gostev, Olga Kalinogorskaya, Ksenia Ivanova, Ekaterina Kalisnikova, Irina Lazareva, Polina Starkova, Sergey Sidorenko

**Affiliations:** 1 Department of Medical Microbiology and Molecular Epidemiology, Pediatric Research and Clinical Center for Infectious Diseases, 194017 Saint Petersburg, Russia; vgostev@niidi.ru (V.G.); kalinogorskaya@niidi.ru (O.K.); xennia55@niidi.ru (K.I.); katrinsp2006@niidi.ru (E.K.); partina-irina@niidi.ru (I.L.); sps_96@niid.ru (P.S.); 2 Department of Medical Microbiology, North-Western State Medical University named after I.I Mechnikov, 195067 Saint Petersburg, Russia

**Keywords:** MSSA, in vitro selection, beta-lactam resistance, oxacillin, ceftaroline, meropenem whole genome sequencing, penicillin-binding proteins

## Abstract

Selective pressure of beta-lactams is thought to be responsible for mutation selection in methicillin-susceptible *Staphylococcus aureus* (MSSA). We used next-generation sequencing to compare the genomes of beta-lactamase-positive (SA0707) and -negative (SA0937) MSSA isolates with their derivatives obtained after selection with oxacillin, ceftaroline, or meropenem. Selection with oxacillin and ceftaroline caused a rapid and significant (6–8 times) increase in the minimum inhibitory concentration (MICs) of oxacillin, penicillin, amoxicillin/clavulanate, and ceftaroline against the derivatives of both isolates, associated with growth impairment. Selection with meropenem caused a limited increase in the MICs of all beta-lactams against both isolates. During the initial stages of selection (after 5–15 passages), mutations were detected only in some reads, which indicated the heterogeneity of the population; however, during the later stages, either the population reversed to the wild type or fixation of the mutation was observed in the entire population. Selection with different beta-lactams caused diverse mutational events, but common mutations were detected in *gdpP,* all penicillin-binding proteins, cell wall regulators (*vraST, graR*), and deletions in the promoter region of *pbp4*. Therefore, the disk diffusion test with cefoxitin does not reveal resistance associated with these mechanisms in some cases, which can lead to the failure of beta-lactam therapy.

## 1. Introduction

*Staphylococcus aureus* is one of the main pathogens responsible for community-acquired (CA) and hospital-acquired (HA) infections in humans [1,2]. Dissemination of penicillinases among *S. aureus* between the 40s and 60s is the first example of the worldwide spread of antimicrobial resistance [3]; however, it was overcome by the development of penicillinase-stable beta-lactams. In subsequent years, several resistance mechanisms against penicillinase-stable beta-lactams have been identified in *S. aureus*. Methicillin-resistant *S. aureus* (MRSA) is one of the most important multidrug-resistant organisms worldwide, as it is the second most common cause of infections due to antibiotic-resistant bacteria in the European Union and European Economic Area [4] and is a member of “ESKAPE” (*Enterococcus*, *Staphylococcus, Klebsiella, Acinetobacter, Pseudomonas*, and *Enterobacter*), a group of resistant pathogens that pose the greatest threat to the healthcare system [5]. MRSA harbors *mecA* or its homologs (*mecB* and *mecC*) that encode low-affinity penicillin-binding proteins (PBPs), and is resistant to all beta-lactams, except ceftaroline and ceftobiprole. Genes distantly related to *mecA* have been identified in the genomes of other staphylococci and related bacteria, and a guideline for reporting novel homologues has been proposed [6]. Moreover, traditional MRSA circulating in hospitals is referred to as hospital-acquired MRSA (HA-MRSA). New genetic lineages of MRSA have emerged in recent decades, including community-acquired MRSA (CA-MRSA), that cause infections in people without predisposing risk factors, and livestock-associated MRSA (LA-MRSA), which causes infections in livestock animals and increasing number of severe infections in humans [7]. Beta-lactam administration is based on the assessment of the susceptibility of *S. aureus* to cefoxitin, preferably using the disk-diffusion method. The European Committee on Antimicrobial Susceptibility Testing (EUCAST) and Clinical and Laboratory Standards Institute (CLSI) [8,9] state that resistance to cefoxitin correlates with the presence of *mec*-type genes and reliably predicts methicillin resistance, whereas susceptibility of a strain toward cefoxitin predicts its susceptibility to all penicillinase-stable beta-lactams. 

However, *S. aureus* that demonstrates decreased susceptibility to cefoxitin and oxacillin or is susceptible to increased minimal inhibitory concentrations (MICs) of other beta-lactams, but that does not carry the *mec*-type genes, has been detected. Such isolates are commonly referred to as borderline oxacillin-resistant *S. aureus* (BORSA) because oxacillin is widely used for their detection [10]; however, there is currently no consensus on the terminology, as some authors prefer the general term methicillin-resistant lacking *mec* (MRLM) and different terms for the two subgroups depending on the resistance mechanism of the isolates: BORSA in a narrow sense for beta-lactamase hyperproducers (BHPs) and modified *S. aureus* (MODSA) for isolates with resistance due to mutations [11]. Staphylococcal beta-lactamases (BlaZ) are serologically classified into types A to D, and type C BlaZ from BORSA hydrolyzes oxacillin faster than that from methicillin-susceptible *S. aureus* (MSSA) [12]. In MODSA, mutations in the *pbp* gene, in the *pbp4* promoter region, and *gdpP* (c-di-AMP regulator), and *yjbH* (disulfide stress effector) genes are known to increase resistance to oxacillin and other beta-lactams in the absence of *mec*-type genes. The selective pressure of beta-lactams is the most probable mechanism for mutation selection. Adaptive mutations in *S. aureus* exposed to oxacillin in vitro have been well-studied [13]; however, little is known about the ability of other beta-lactams to select mutations and their cross-activity against MODSA isolates. Meropenem administration in patients with cystic fibrosis was shown to drive collateral resistance against ceftaroline in MSSA isolates [14]. In the present study, we used next-generation sequencing to compare the genomes of *blaZ*-positive and -negative MSSA isolates with their derivatives obtained after selection with oxacillin, ceftaroline, or meropenem.

## 2. Results

### 2.1. Phenotypic Changes during Selection with Beta-Lactams

The MICs of oxacillin, amoxicillin/clavulanate, cefoxitin, ceftaroline, and meropenem against the parental isolates SA0707 and SA0937 were below the EUCAST epidemiological cut-off (Table 1). Parental SA0707 isolate demonstrated resistance to erythromycin, tetracycline and penicillin and carries corresponding resistance genes (*ermA, tetK* and *blaZ*) (Supplementary Material Table S1). During the selection with beta-lactams, no changes in susceptibility to erythromycin, tetracycline, and corresponding genes in SA0707 isolate were detected; however, loss of the plasmid carrying *blaZ* and *qacB* has been noted. Isolate SA0937 was susceptible to all non-beta-lactam antibiotics tested and did not contain resistance genes, and no changes were detected during selection (Supplementary Material Table S1).

Selection with oxacillin and ceftaroline led to a rapid and significant (6–8 times) increase in the MICs of oxacillin, penicillin, amoxicillin/clavulanate, and ceftaroline against the derivatives of both isolates. We were unable to determine the exact MIC values against the SA0937 derivatives during selection because after ten passages, the oxacillin derivatives lost the ability to grow in Mueller-Hinton medium, but retained the ability to grow in brain heart agar. After ten passages in antibiotic-free brain heart agar, the derivatives restored the ability to grow in Mueller-Hinton medium and partially lost the acquired resistance. Derivatives of the SA0707 isolate did not lose the acquired resistance after passaging on antibiotic-free medium. The increase in the MICs of cefoxitin and meropenem during selection on oxacillin and ceftaroline was insignificant (not more than 2–5 times). Despite the development of resistance based on the broth dilution method, the derivatives of both isolates retained susceptibility to cefoxitin according to the disk diffusion method.

Selection with meropenem led to a limited increase in the MICs of all beta-lactams against both isolates; the acquired resistance was stable and did not revert after ten passages in antibiotic-free medium. Derivatives of SA0707 and SA0937 isolates obtained after 15 and 30 passages, respectively, demonstrated reduction of cefoxitin zone inhibition diameter by the disk diffusion method below the breakpoint.

**Figure 1:**
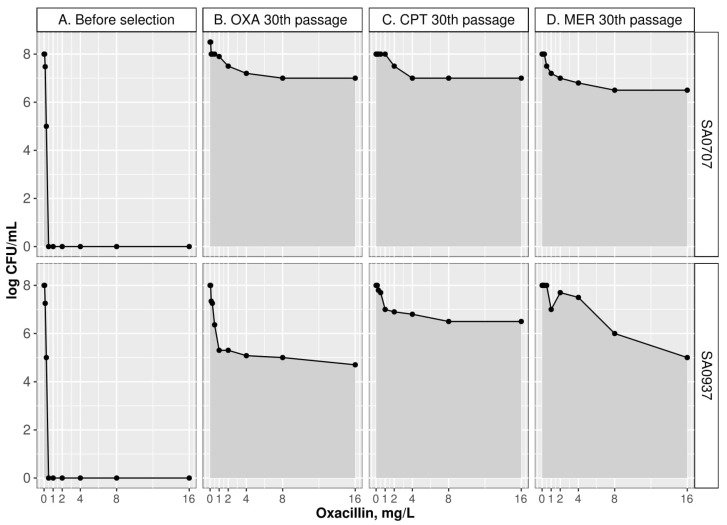
Population analysis profile (PAP) of parental isolates and their derivatives after 30 passages against oxacillin (OXA), ceftaroline (CPT), and meropenem (MER).

Population analysis profile (PAP) revealed a significant increase in the area under the curve (AUC) of the derivatives (AUC_derivatives_) compared with the parental isolates (AUC_wt_). After 30 passages, the AUC_derivatives_/AUC_wt_ ratio was higher than 2 in all cases (Table 1). Results of PAP are illustrated in Figure 1. After passage 30, the derivatives of both isolates were characterized by heterogeneity in the level of susceptibility to oxacillin; they contained subpopulations with both low and high levels of resistance. In the derivatives of the SA0937 isolate, this heterogeneity was more pronounced.

Growth kinetics of parental isolates was compared to those of derivatives obtained after 30 passages (Table 2). Both parental isolates demonstrated similar parameters of growth kinetics with a difference revealed only in the duration of the lag phase, which was slightly longer for isolate SA0707. The most pronounced growth impairments (decrease in growth rate, increase in doubling time, and lag phase duration) were observed in SA0937 derivatives selected by all beta-lactams. A significant impairment in SA0707 derivatives was detected only after selection by oxacillin. For a better illustration of the described patterns, growth curves are shown (Supplementary Material, Figure S1).

Changes in autolytic activity were identified after selection (Table 2). Autolysis was considered as a strain variable. Isolate SA0937 was autolyzed under the influence of triton before selection, whereas isolate SA0707 was resistant. No significant changes in autolysis were detected for the SA0707 isolate due to selection on the three beta-lactams. However, there was an increase in autolysis in SA0937 cells after selection on oxacillin. Despite this, it is impossible to clearly interpret these results because the cells of the isolate were lysed, even with the addition of phosphate-buffered saline (PBS) due to an unstable phenotype with impaired fitness.

### 2.2. Genetic Changes during Selection

The entire cell biomass that grew on the petri dishes with antibiotics was used for sequencing, and the percentage of reads with different mutations was determined. During the selection process, various pathways for mutations were observed. In most cases, at the initial stages of selection (after 5–15 passages), mutations were detected only in some of the reads, which indicated the heterogeneity of the population. At the subsequent stages of selection, either the reversion of the population to the wild type (WT) or the fixation of the mutation in the entire population was observed. The dynamics of the mutation are presented in the Supplementary materials (Table S2). Synonymous mutations were excluded from the analysis.

#### 2.2.1. Genetic Changes during Selection with Oxacillin

A 91 bp deletion was detected in the *pbp4* promoter region of both strains, which appeared after the 5th and 30th passages in SA0937 and SA0707 strains, respectively (Table 3). The SA0707 strain also had a minor mutation—E297K (21.8% of reads)—in *pbp4*; however, this substitution was not detected after 10 passages on antibiotic-free medium. Both strains had mutations in the *gdpP* gene: in the SA0937 strain, at the 5th passage, 23% of reads revealed a 1 bp deletion with subsequent distribution among 100% of reads, and in strain SA0707, a minor mutation Y475C was observed (7% of the reads). In SA0937, after 40 passages in antibiotic-free medium, mutation L81I in DacA, which synthetizes c-di-AMP, was identified in 77% of the reads. Mutations in *vraT* appeared by the 15th passage in SA0707 in 100% of reads, and by the 5th passage in 21% of the reads in strain SA0937 with subsequent fixation in the population. Other genetic events that were detected varied in both strains.

Different mutations were identified in the cell wall (CW) stimulon in both derivative strains: in the SA0937 derivative, mutations were observed in the *vraS* and *stp1* genes, and in the SA0707 derivative, in the *graR* gene. In addition, the mutation in *pbp2* and the 117-bp deletion in the *murAB* promoter region from the 30th passage were detected in the SA0707 derivative strain. Additionally, the appearance of several (A95P, P192R, A384V) minor amino acid substitutions (22–38.5% of reads) in MurAB were detected after subculturing on antibiotic-free medium. The derivative of the SA0707 strain was characterized by the presence of a large deletion (22,458 bp), which appeared at the 40th passage (passage on antibiotic-free medium) and included 29 genes (coordinates in the SACOL reference genome: 1,353,558–1,376,106 bp). This deletion region contained the *cls1* cardiolipin synthase gene and genes involved in L-threonine biosynthesis, as well as genes with unknown functions. Mutations were also identified in SA0707 in the *mprF*, *fabZ*, *pcrB*, and *lgt* genes, which are responsible for the biosynthesis of membrane phospholipids.

Mutations in genes involved in wall teichoic acid (WTA) biosynthesis were detected in both derivative strains. At the 15th passage, a 236 bp deletion in *dltD* was detected in SA0937 cells. The stop codon at position L323* was found in *tagO* after 15 passages in SA0707 and, at the 5th passage, a minor mutation (18% of reads) in the *fmtA* gene, which is involved in the alanylation of teichoic acids, was also detected. Mutation in *guaA* (G109C) was observed after 15 passages in strain SA0937.

#### 2.2.2. Genetic Changes during Selection with Ceftaroline

Deletions in the *pbp4* promoter region were detected in both derivative strains during ceftaroline treatment (Table 3). This mutation was initially identified in 30% of SA0937 reads by the 30th passage; however, at the 40th passage, the deletion was fixed in the whole cell population (100% of reads). Mutations in Pbp4 (T201A and N138I) were detected at the 30th passage (20% of reads) in the same strain, and by the 40th passage, they were confirmed among 100% of reads. Moreover, an additional mutation was detected in *pbp1* after the 5th passage in SA0937 cells. At the 15th passage, in the SA0707 derivative strain, a 91 bp deletion was detected in the *pbp4* promoter region at the same position as that found after selection on oxacillin. This isolate also demonstrated a 171-bp deletion in the *murAB* promoter region after the 15th passage. A similar deletion was detected during the selection of oxacillin. Mutations were also identified in *gdpP* of the SA0937 strain: a double mutation at the 5th passage at the H621Y position (30% of reads) and in the stop codon at the R540* position (70% of reads). The last mutation was fixed in the population and was detected in all subsequent passages.

At the 40th passage on antibiotic-free medium, a mutation in DacA (E228G) was observed. Both derivative strains showed mutations in the genes involved in the CW stimulon. In particular, at the 40th passage on antibiotic-free medium, deletions in *graR* and *vraS* were detected in SA0707 and SA0937 derivative strains, respectively. A 1-bp deletion was identified in strain SA0937 in the monofunctional peptidoglycan glycosyltransferase gene (*sgtB*) after the 5th passage.

Besides mutations in genes associated with beta-lactam resistance, genetic events in other bacterial cell systems were also identified. For instance, in the SA0937 derivative strain, mutations were detected in the genes responsible for aerobic respiration *cydA*, teichoic acid biosynthesis-*dltA*, *tagA*, general metabolism and DNA repair systems, and transcription (*greA, ruvB,* and *mutL*). Mutations in the global regulator Spx (stop codon at Q122* position), VraD, and FadE, which are involved in fatty acid metabolism, were also identified at different stages of ceftaroline selection.

Strain SA0707 was characterized by a lower number of mutational events than SA0937 during ceftaroline selection. Mutations in the SA0707 derivative were detected in *tagO*, *mpsB*, some other genes of general metabolism, and genes with unknown functions. The stop codon at position K426* of OatA was identified.

#### 2.2.3. Genetic Changes during Selection with Meropenem

*gdpP* mutations were observed in the isogenic strains after selection on meropenem (Table 3). Numerous mutations in *pbp1* were identified in the early stages of selection in both derivative strains. In the SA0937 derivative strain, mutations were detected in all penicillin-binding proteins (*pbp1, pbp2, pbp3, and pbp4*), but in the SA0707 derivative strain, mutations were detected only in *pbp1* and *pbp2*. Minor mutations were observed in *vraT* after 5th passage in the SA0707 derivative.

The mutation in *tagH*, which is responsible for teichoic acid biosynthesis, was found in strain SA0707. In addition, mutations in the lipid metabolism gene (*accD*), gene encoding for foldase (*prsA*), and other genes associated with general metabolism were identified in this strain.

Mutations in the CW stimulon were detected in the SA0937 derivative strain in *vraS* at the 15th passage, in *dltA*, which is involved in the biosynthesis of teichoic acids, and in *gpsB*, which is involved in the formation of septum during cell division. Another mutation affecting cell division was found in the SA0937 derivative strain, in *smc*, which is involved in chromosome segregation. Mutation A211V in DacA (77% of reads) was found after 40 passages on antibiotic-free medium in the SA0937 derivative strain. In addition, mutations involved in various bacterial metabolic pathways were identified, such as *acuC* (acetoin utilization), sugar metabolism (*ccpN, cscA, scrA*), and other systems.

## 3. Discussion

Methicillin-susceptible *mecA*/*mecC*-negative *S. aureus* strains with decreased susceptibility to beta-lactams are distributed worldwide. BORSA isolates cause HA and CA infections with varied localization; their exact prevalence is difficult to establish due to the need for special detection methods. It is reported that the BORSA phenotype can be detected in 1.4–12.5% of *S. aureus* isolates [10,11,15]. Despite many studies, decreasing susceptibility against beta-lactams in MSSA is not fully understood. Here, we focused on the possible mechanisms of resistance in MSSA via in vitro selection with three beta-lactam antibiotics widely used in clinical practice. Two strains, *blaZ*-positive and *blaZ*-negative, were included in the selection study. After selection, both strains were characterized by high levels of resistance and cross-resistance to beta-lactams, including ceftaroline, regardless of the antibiotic used for selection. The phenomenon of cross-resistance during selection with antibiotics and disinfectants has previously been reported. A recent study showed that meropenem promoted resistance in MRSA against ceftaroline [14]. A decrease in susceptibility to oxacillin and mutations were observed in *S. aureus* after prolonged treatment with subinhibitory concentrations of sodium hypochlorite [16]. 

A significant portion of the mutations identified during selection are localized in genes known to be involved in resistance. Moreover, in a recent study [13], mutations in c-di-AMP signal transduction pathways, chaperone-protease complexes (*clpX, clpP*), and CW regulators (*vraRS* and *graRS*) were recognized as the main mechanism of low-level oxacillin resistance. Mutations in *pbp3* were also identified, but they were not linked with oxacillin resistance in site-directed mutagenesis. The authors also indicated that β-lactamase (*blaZ*) might facilitate or stabilize oxacillin resistance. Indeed, in the present study, the strain SA0937, which lacked *blaZ*, demonstrated accumulation of many more mutations during passaging against all antibiotics, characterized by severe fitness cost compared with those in SA0707. The loss of the plasmid harboring *blaZ* in strain SA0707 was observed after selection on oxacillin and meropenem but not after selection on ceftaroline. The *blaZ*-plasmids have been reported to be stable in MRSA genomes after selection on ceftaroline [17].

On one hand, it is not difficult to develop resistance to oxacillin, which is comparable with accumulation of resistance to rifampicin or ciprofloxacin, as demonstrated by Giulieri et al. [13] On the other hand, oxacillin selection has a strong influence on fitness, which is reflected in the dramatic decrease in growth rate observed in the current study. Moreover, the SA0937 strain lost the ability to grow on Mueller-Hinton medium probably due to mutations in the genes responsible for general metabolism. After passaging, both derivative strains demonstrated increased oxacillin MIC and an increase in the AUC_derivatives_/AUC_wt_ ratio. However, the SA0937 derivative strain was unstable, and after ten passages on antibiotic-free medium (after selection on oxacillin and ceftaroline), a decreased in oxacillin MIC, and AUC_derivatives_/AUC_wt_ ratio was observed. Both derivative strains demonstrated a decreased growth rate after selection on the three beta-lactams.

Derivative strains were characterized by diverse mutational events, but common mutations also occurred. The following mutations were common in both strains, including mutations in *gdpP*, all PBPs, CW regulators (*vraST, graR*), and deletions in the promoter region of *pbp4* were common in both isolates. The majority of single nucleotide polymorphisms (SNPs) in *pbp1* and *pbp2* were identified after selection on meropenem. All these genes were associated with beta-lactam resistance. For instance, mutations in *pbp1* and *pbp2* are associated with the BORSA phenotype and have been described in clinical isolates [11]. Mutations in PBP4 or its promoter are correlated with beta-lactam resistance, including ceftaroline [18]. Many studies have shown the role of SNPs in genes of c-di-AMP metabolism in the development of resistance to cell wall-targeting agents [19]. In SA0937, mutation in *stp1*, which is associated with beta-lactam resistance, was identified during early passages [20]. During selection, mutations in genes involved in WTA biosynthesis were also identified: *tagOHA, dltAD*, and *sgtB*. The role of WTA in resistance to beta-lactam is not fully understood; however, it has been documented that changing the expression profile and mutations causes change in the level of D-alanylation of teichoic acids and the spatial structure of the CW [21]. These changes may have additional effects on antibiotic resistance. Moreover, in a study by Karinou et al. [22], inactivated SgtB promoted a 4-fold increase in the MIC of oxacillin. *dltABCD* regulates the activity of the autolytic system, since a decrease in the positive charge of the CW accelerates autolysin activity and is linked to cationic antimicrobial peptide resistance and daptomycin [23].

SA0707 mutations in *murAB* and deletions in its promoter were identified after selection on oxacillin and ceftaroline. MurAB catalyzes the early stages of assembly of peptidoglycan monomers in the cytoplasm [24]. The role of this protein in resistance is unknown. A stop codon in O-acetyltransferase (*oatA*), associated with lysozyme resistance, was detected in the derivative strain of SA0707 after selection on ceftaroline, and deletion in this gene was observed after ceftaroline selection in MRSA [17].

In addition to mutations in the listed genes, we found many mutations in the genes of different metabolic pathways that had not previously been associated with the formation of resistance. These mutational events were strain-specific, and their role in resistance development should be confirmed with additional studies. The role of mutations in genes involved in fatty acid biosynthesis (*fabZ, accD, fadE*), which have been identified in derivative strains, is not clear. Interestingly, in the SA0707 derivative strain, after passaging on oxacillin, a large deletion was identified; the deletion region included cardiolipin synthase *cls1*, a part of membrane phospholipid biosynthesis that is involved in daptomycin resistance [25]. However, the SA0707 derivative strain was completely susceptible to daptomycin in all passages. During selection, mutations in different regulatory genes (*spx, rpoC, agrA*) and different metabolic pathways, which are part of general metabolism, were identified. Mutations in genes involved in DNA metabolism and repair (*ruvB*, *mutL*) were also identified. Probably, these mutational events could play a compensatory role. That these genes may be directly involved in the formation of resistance is also possible, as in a recent study it was shown that mutations in underappreciated noncanonical genes, such as those related to central carbon and energy metabolism, are implicated in antibiotic resistance [26].

Allele frequency detection approaches were used for SNP calling in the whole genome sequencing of the derivative strains. This approach allows the detection of minor or low frequency reads with mutations (“heteromutations”) and, therefore, detects subpopulations. Such an approach is used to study population fluctuations in long-term evolution experiments [27]. In the present study, during beta-lactam selection, some mutational events were initially covered by minor counts of reads; however, these events were subsequently fixed in the total population or eliminated. It is likely that during selection, a minor subpopulation is initially generated and then leads to clonal expansion. For instance, considering mutations in the CW regulon, in the SA0937 derivative strain, at the 5th passage on oxacillin, < 30% of reads had mutations in *the vraT, gdpP, stp1*, and *pbp4* promoters. However, by the 15th passage, all these mutations occurred in 100% reads. In another example, in the SA0707 derivative strain during meropenem selection, multiple mutations were observed in *pbp1*; at the 5th passage, substitution W351K was detected in 57% of the reads, but this substitution was eliminated. However, mutations A482V + L18F and heteromutation H375D were identified between 15 to 40 passages in *pbp1*. The level of heteromutations was higher in the SA0937 strain, which coincides with the results of PAP. The accumulation of mutations in derivative isolates led to a fitness cost, which manifested as a growth impairment.

In the current study, an important finding was that in the disk-diffusion method with cefoxitin, only the derivatives after selection on meropenem demonstrated a resistant phenotype with a low diameter inhibition zone. Such an effect may be linked to accumulation mutations in almost all PBPs (Pbp1 to Pbp4). This is dangerous because such phenotypes could be missed during routine susceptibility screening and can be affected by inappropriate beta-lactam indications. Thus, susceptibility tests should be performed using several beta-lactam antibiotics.

Treatment regimens for infections caused by BORSA isolates have not been standardized, and evidence is accumulating that traditional beta-lactams are not effective [28], which justifies the use of antibiotics of other groups (glycopeptides, lipoglycopeptides, lipopeptides, oxazolidinones and others) despite the trend towards development of resistance [29,30]. Our data indicate that selection by oxacillin or meropenem leads to cross-resistance to cephalosporin with increased anti-MRSA activity ceftaroline.

The current study had a few limitations. The small number of strains included in the study reflected on the interpretation of the received data, and it cannot be ruled out that observed mutational events are not associated with resistance but are strain-specific. The study did not include gene expression or transcriptomic assays. The detected mutations were not confirmed by site-directed mutagenesis. Many of the genetic events that include general metabolism, fatty acid biosynthesis, and WTA biosynthesis pathways should be explored in future studies. 

## 4. Materials and Methods

### 4.1. Bacterial Strains and Susceptibility Testing

Two methicillin-susceptible *mecA*-, *mecB*-, and *mecC-negative S. aureus* strains (SA0937 *blaZ*-negative, single locus variant of sequence type (ST) 97, and SA0707 *blaZ*-positive, belonging to ST8) were recovered from healthy carriers in Moscow in 2016. The MICs of oxacillin, penicillin, amoxicillin/clavulanate, cefoxitin, ceftaroline, and meropenem (Molekula, Darlington, UK) were determined according to ISO 20776-1 (2006) and interpreted according to the EUCAST recommendations. 

### 4.2. Multistep Resistance Selection

Strains SA0937 and SA0707 were serially passaged on Brain Heart Infusion (BHI) agar plates containing increasing concentrations of oxacillin, ceftaroline, or meropenem (Supplementary material Figure S2). At the first stage, suspensions of the isolates (10^9^ CFU/mL) were streaked onto plates with two-fold dilutions of each antibiotic from 1 to 4-fold MIC and incubated at 37 °C for 72 h. For the next passage, colonies from the plates with the highest concentration of each antibiotic were used. If the colonies appeared more than 18 h post-inoculation, the cell suspension was transferred to new plates containing the same concentration range of antibiotic as in the previous round. However, if the time taken for the colonies to appear was less than 18 h, the concentration range of the antibiotic was doubled. MIC measurements, PAP, and whole genome sequencing were performed before selection, after 5, 15, and 30 passages with antibiotics and after 10 passages on antibiotic-free medium (40th passage).

### 4.3. PAP

PAP was performed according to the microdilution modification proposed in a previous study [31]. Four dilutions (10^−1^, 10^−3^, 10^−5^, and 10^−7^) of the initial suspension (10^8^ CFU/mL) of each strain were prepared. Three 10-µL droplets of each dilution were plated on oxacillin-containing BHI agar plates (0.06, 0.125, 0.25, 0.5, 1.0, 2.0, 4.0, 8.0, and 16.0 mg/mL). The inoculated plates were incubated for 48 h at 37 °C. Plated droplets containing 5–50 CFU were selected for counting, and the average number of colonies per oxacillin concentration was determined. Plots showing the number of CFUs in the presence of each concentration of oxacillin were constructed. AUC was calculated using the R base package. The ratio of AUC for derivative isolates to AUC for parental (WT isolate) (AUC_derivative_/AUC_WT_) was calculated.

### 4.4. Measurement of Growth Rates

The optical density of the growing cultures was measured at 600 nm (OD_600_) every 10 min using an Infinite 200 Pro plate reader (Tecan, Grödig, Austria). Growth curves were analyzed using the R package, Growthcurver [32]. The growth rate (r, min^−1^) and doubling time (Dt, min) were calculated. The lag time was determined as the time from inoculation to the first increase in OD_600_.

### 4.5. Measurement of Induced Aautolytic Activity

The induced autolytic activity was measured as previously described [33]. Briefly, cells were grown to an OD_600_ of 0.7, and chilled on ice. They were then washed twice with ice-cold (PBS, pH = 7.4) and resuspended to an OD_600_ of 1.0, in PBS supplemented with 0.05% Triton X-100. The cells were incubated in a 96-well plate at 30 °C and shaking at 142 rpm (amplitude 6 mm), and the rate of autolysis was assessed by measuring OD_600_ every 10 min for a period of 5 h using a plate reader. The percentage of lysed cells was then calculated. 

### 4.6. Whole Genome Sequencing

Genomic DNA was extracted using a PureLink™ Genomic DNA Mini Kit (Invitrogen™, Waltham, MA, USA) and preliminary cell lysis was performed with 1 mg/mL lysostaphin (Sigma-Aldrich, St. Louis, MO, USA). A Nextera XT or Nextera Flex Kit (Illumina, San Diego, CA, USA) was used for DNA library preparation, followed by sample indexing and amplification according to the manufacturer’s protocol. Concentrations and fragment size of DNA libraries were validated with a Qubit Fluorometer using dsDNA HS Assay Kit (Invitrogen, USA) and 4150 TapeStation System (Agilent, Santa Clara, CA, USA) with High Sensitivity DNA ScreenTape kits, respectively. DNA libraries were sequenced using a MiSeq instrument (Illumina, USA). 

### 4.7. Bioinformatic Analysis

The reads were filtered and trimmed using trimmomatic [34]. De novo contigs were assembled using the SPAdes [35]. Reads of the derivatives were aligned onto assembled contigs of WT isolates and onto the reference genome COL (CP000046.1) using Bowtie and SAMtools [36,37]. Read allele frequency was detected using the mixed allele model available in Breseq software [38]. The threshold was set at 5%, and all mutational events below this point were excluded. Sequence reads of strains before selection were aligned against themselves, and the detected SNPs were excluded from all data to prevent the identification of false positive mutations due to assembly of reference genome errors. All repeat regions, mobile genetic elements, phage-associated loci, and adhesins with homopolymer nucleotide areas were excluded from the analysis due to the high rate of nonspecific polymorphisms.

## 5. Conclusions

Resistance to all beta-lactams including ceftaroline in *mecA*-negative *S. aureus* could be develop through selection on any beta-lactam due to mutations in *gdpP,* all PBPs, CW regulators (*vraST, graR*), and deletions in the promoter region of *pbp4*. The resistance develops through the formation of mixed populations consisting of clones with specific mutations and clones without mutations (heteroresistance). The disk diffusion test with cefoxitin does not allow for identification of resistance associated with these mechanisms in many cases, which can lead to the failure of beta-lactam therapy.

## Figures and Tables

**Table 1:** Susceptibility features of parental strains and their isogenic derivatives.

Isolate	Ab Selection	Passage	OXA	CPT	MER	FOX	AMC	PEN	FOX DDM, mm	PAP (AUC_derivative_)/(AUC_WT_)
	ECOFF *		≤2	≤0.5	≤0.5	≤4	-	≤0.125	22≤	-
SA0937	WT	0	0.125	0.25	0.06	4	0.25	0.06	30	-
	OXA	5	16	4	0.25	8	16	8	32	2.24
	OXA	15	ND **	ND	ND	ND	ND	ND	27	2.43
	OXA	30	ND	ND	ND	ND	ND	ND	22	2.31
	Ab free	40	2	1	1	8	1	0.25	23	1.93
	CPT	5	32	16	0.5	2	32	32	27	2.4
	CPT	15	32	16	1	4	16	32	28	2.62
	CPT	30	64	128	2	8	32	64	24	2.69
	Ab free	40	2	4	0.5	8	4	2	28	1.48
	MER	5	4	1	1	8	2	1	28	2.05
	MER	15	8	4	2	8	2	4	24	2.55
	MER	30	8	4	8	16	2	4	0	2.75
	Ab free	40	8	2	8	16	2	4	14	2.49
SA0707	WT	0	0.5	0.25	0.06	4	2	32	28	-
	OXA	5	4	0.5	0.25	2	2	16	27	1.55
	OXA	15	32	2	1	2	32	64	29	2.73
	OXA	30	32	16	2	8	32	64	25	2.85
	Ab free	40	32	32	2	8	16	32	24	2.68
	CPT	5	1	1	0.5	4	4	128	22	1.08
	CPT	15	32	32	0.5	8	32	32	24	2.3
	CPT	30	128	64	2	16	64	128	22	2.82
	Ab free	40	128	64	2	16	64	128	22	2.73
	MER	5	2	1	1	8	4	16	28	1.9
	MER	15	8	2	4	4	4	16	14	2.37
	MER	30	8	2	4	8	4	4	0	2.71
	Ab free	40	8	2	4	8	4	4	14	2.52

Abbreviations: WT, wild type strain; Ab free, passages on antibiotic-free medium after resistance selection; OXA, oxacillin; CPT, ceftaroline; MER, meropenem; FOX, cefoxitin; AMC, amoxicillin and clavulanic acid; PEN, penicillin, FOX DDM, disk-diffusion method with FOX; PAP, population analysis profile. * According to EUCAST data (https://mic.eucast.org/) accessed on 31 March 2021; ** ND—not-detectable results; derivative isolate SA0937 after selection on oxacillin could not grow in Muller-Hinton media.

**Table 2:** Phenotypic features of parental and isogenic derivatives.

Figure	Strain	Wild Type	Antibiotics Used for Selection		
			OXA	CPT	MER
**Growth Kinetics**					
Dt, min	SA0937	27.25 (26.86–27.47)	41.30 (39.0–42.6)	33.53 (33.07–34.0)	36.81 (34.2–39.5)
	SA0707	25.42 (25.08–26.25)	52.32 (51.01–53.68)	38.31 (37.56–39.14)	28.89 (28.02–31.23)
r, min^−1^	SA0937	0.025 (0.025–0.026)	0.016 (0.016–0.017)	0.02 (0.02–0.02)	0.018 (0.017–0.02)
	SA0707	0.027 (0.026–0.027)	0.013 (0.012–0.014)	0.018 (0.017–0.019)	0.02 (0.019–0.021)
Lag, min (Range)	SA0937	69–79	158–168	128–138	128–138
	SA0707	89–99	227–237	168–178	168–178
**Induced Autolysis**					
OD_600_/lysed cells, %	SA0937	55.8 (50.5–62.0)	68.15 (62.4–75.3)	35.0 (29.5–39.7)	58.6 (53.6–64.1)
	SA0707	14.0 (7.0–16.4)	8.0 (2.0–13.0)	14.4 (10.2–18.6)	16.3 (10.0–19.4)

Notes: Dt (doubling time), median (M), and interquartile range (Q1-Q3) are shown; r, growth rate; Lag, lag phase of kinetic growth; OXA, selection on oxacillin; CPT, selection on ceftaroline; MER, selection on meropenem; OD600/lysed cells, percentage of lysed cells.

**Table 3:** Mutations associated with CW regulon andWTA beta-lactam resistant derivative strains.

		SA0937 (*blaZ*-), Selection on:			SA0707 (*blaZ*+), Selection on:		
	Proteins	OXA	CPT	MER	OXA	CPT	MER
CW	Pbp1	-	H499R	G408V, W351L, W351R	-	-	A482V, P431L, H375D, W351K, L18F
	Pbp2	-	-	I19N, G587S, M559I, T552I, A416E, G142S	A450D	T552I, A416T	-
	Pbp3	-	-	G286V, S634F	-	-	-
	Pbp4	P ∆91 bp	P ∆1 bp, T201A, N138I	F241L, N141T	P ∆91 bp	P ∆91 bp	-
	GdpP	∆1 bp	H621Y, R540Stop	E108Stop	Y475C	-	R289C
	VraS	T274K	∆1 bp	C60Y	-	-	-
	VraT	P174Q	-	-	W119R	-	G226V
	MurAB	-	-	-	P ∆171 bp	P ∆171 bp	-
	GraR	-	-	-	G59E	∆161 bp	-
WTA	TagA	-	G171E	-	-	G171E	-
	TagH	-	-	-	-	-	Q65Stop
	TagO	-	-	-	L323Stop	L41I	-
	DltA	-	-	-	-	A184V	∆1 bp
	DltD	∆236 bp	-	-	-	-	-
	SgtB	-	-	-	-	∆1 bp	-

All mutations detected at different time points during selection and included minor mutations with allele frequency in range 10–95% are shown. P ∆-deletion in promoter regions, ∆-deletion in coding region; CW, genes involved in cell wall biosynthesis; WTA, genes involved in wall teichoic acids biosynthesis.

## Data Availability

Genomic data have been deposited in the NCBI Sequence Read Archive (SRA), and all reads are available from BioProject PRJNA721282.

## Supplementary Materials

The following are available online at https://www.mdpi.com/article/10.3390/antibiotics10060637/s1, Figure S1: growth curves of parental strains and derivatives; Figure S2: Selection schemes; Table S1: All mutational events and allele frequencies detected at different time points of selection on three antibiotics; Table S2. The dynamics of mutation during selection by betalactam antibiotics.
