# Supplementary material for: In Vitro Selection of High-Level Beta-Lactam Resistance in Methicillin-Susceptible Staphylococcus aureus
Source: Antibiotics (Basel). 2021 May 26;10(6):637. doi: 10.3390/antibiotics10060637 (PMC8227848; doi:10.3390/antibiotics10060637)
Supplement: Supplementary file 1 [file antibiotics-10-00637-s001.zip › Supplementary material Figure_S1.pdf]

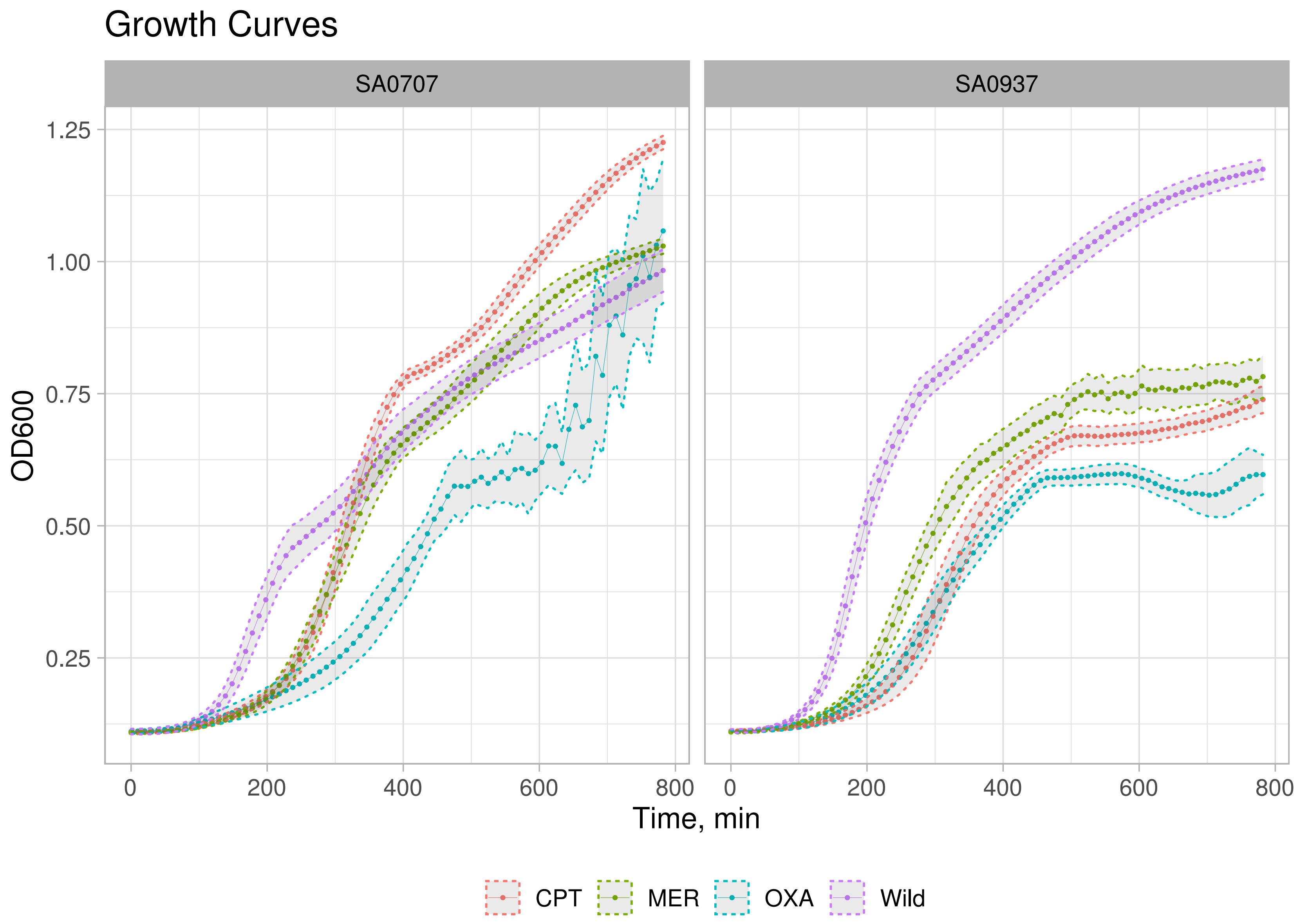
Growth Curves
SA0707
SA0937
OD600
1.25
1.00
0.75
0.50
0.25
0
200
400
600
800
Time, min
CPT
MER
OXA
Wild

Figure S1. Growth curves of parental strains and derivatives after beta-lactam selection

Growth curves of parental strains (Wild) and their derivatives after selection on oxacillin (OXA), ceftaroline (CPT) and meropenem (MER).
