# Supplementary material for: In Vitro Selection of High-Level Beta-Lactam Resistance in Methicillin-Susceptible Staphylococcus aureus
Source: Antibiotics (Basel). 2021 May 26;10(6):637. doi: 10.3390/antibiotics10060637 (PMC8227848; doi:10.3390/antibiotics10060637)
Supplement: Supplementary file 1 [file antibiotics-10-00637-s001.zip › Supplementary material Table S1.pdf]

Table. S1. Antimicrobial susceptibility of parental SA0707 and SA0937 isolates

Isolates	Parameter	Antibiotics															
		CIP	LNZ	ERY	CLI	TGC	VAN	RIF	DAP	GEN	MUP	MXF	TET	FUS	SXT	PEN	Other
SA0707 – WT	MIC, mg/L	<0,25	0,5	>128	<0,06	0,03	0,5	0,03	0,25	0,25	1	<0,125	16	0,25	0,06	32	-
	Gene	-	-	*ermA*	-	-	-	-	-	*ant(9)-Ia*	-	-	*tetK*	-	-	*blaZ*	*qacB*
SA0937 –WT	MIC, mg/L	<0,25	2	0,25	<0,06	0,06	1	<0,004	0,25	1	1	<0,125	0,125	0,125	0,06	0,06	-
	Gene	-	-	-	-	-	-	-	-	-	-	-	-	-	-	-	-

Notes: CIP – ciprofloxacin, LNZ – linezolid, ERY – erythromycin, CLI – clindamycin, TGC – tigecycline, VAN – vancomycin, RIF – rifampicin, DAP – daptomycin, GEN – gentamycin, MUP – mupirocin, MXF – moxifloxacin, TET – tetracycline, FUS – fusidic acid, SXT - trimethoprim/sulfamethoxazole.
