# Supplementary figures and images for: In Vitro Selection of High-Level Beta-Lactam Resistance in Methicillin-Susceptible Staphylococcus aureus
Source: Antibiotics (Basel). 2021 May 26;10(6):637. doi: 10.3390/antibiotics10060637 (PMC8227848; doi:10.3390/antibiotics10060637)

# Selection steps

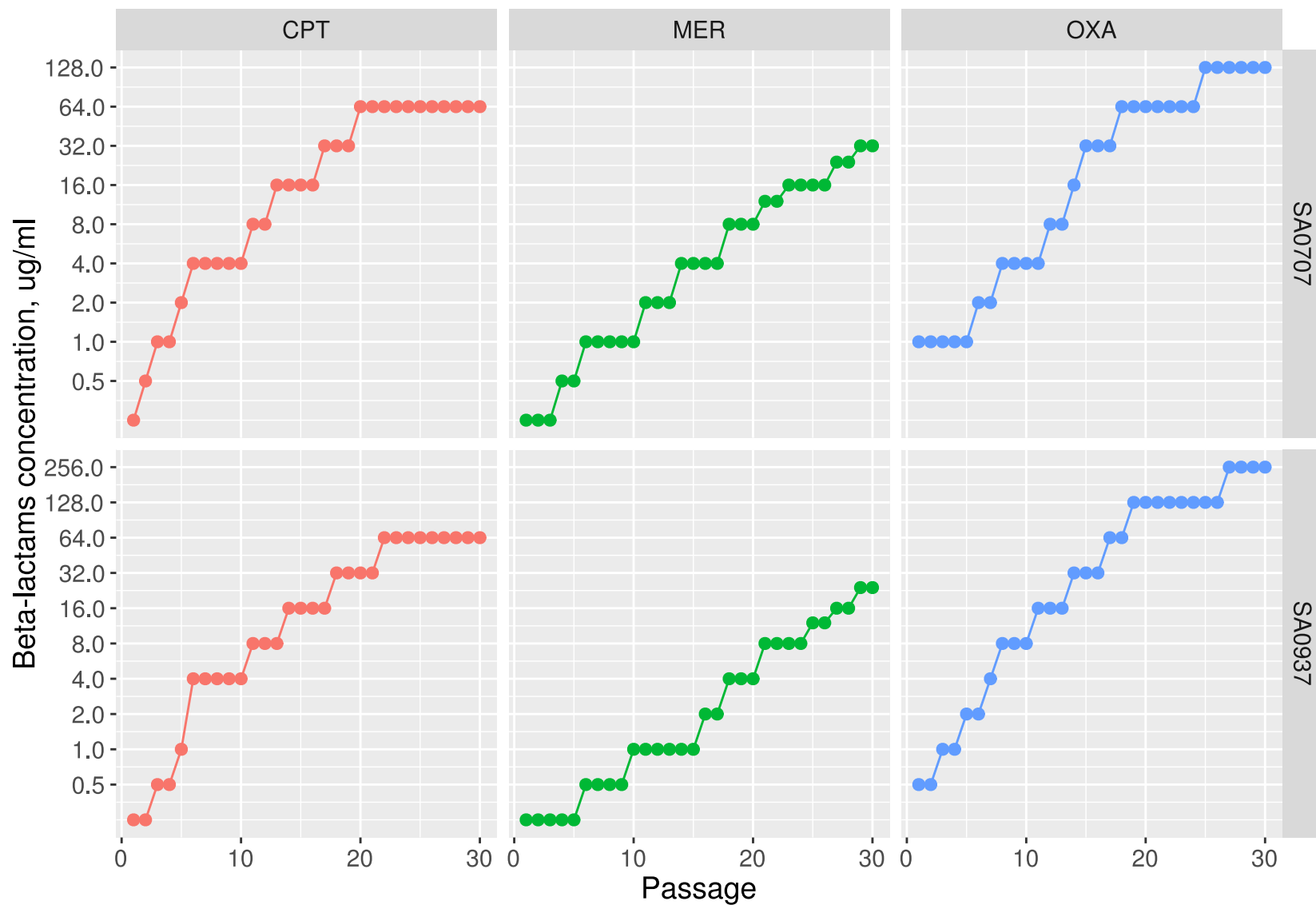

Figure S2. Selection schemes

Supplement: Supplementary file 1 [file antibiotics-10-00637-s001.zip › Supplementary material Figure_S2.pdf]
